# Extraction of Teeth

**Published:** 1888-01

**Authors:** A. Berry

**Affiliations:** Cincinnati


### Extraction of Teeth.

BY A. BERRY, D.D.S., CINCINNATI.

[Read before the Ohio State Dental Society, Springfield, Ohio, October, 1887.]

The extraction of teeth, sometimes very difficult, and often one of the most painful of surgical operations, requires a high degree of skill for its suitable execution.

On this subject Dr. Koecker says: "When we consider the frequent necessity for this operation, and its beneficial effects, even so far as it regards its physical influences, the great importance of it seems to be placed beyond any doubt; and, indeed, on all accounts, it must be allowed that there is not an operation in any branch of surgery more worthy of the particular consideration of the liberal-minded and scientific surgeon, than that which is the subject of our present remarks."

Prof. Taft remarks, "The extraction of teeth is an important operation, requiring for its proper performance, skill, judgmènt, and experience, as well as an accurate knowledge of the parts to be operated upon."

When a patient desires the removal of a tooth an examination is in order, to decide as to the propriety of complying with the request. If there is a probability of saving an important tooth by treatment the patient should be made aware of it. If extraction is still desired it is generally well to remove the tooth unless its retention is very urgent. Usually under these conditions, the patient having little faith or patience, treatment will prove troublesome, and if successful the fee will be grudgingly paid, and the benefits obtained not properly appreciated; and if annoyances ensue, the dentist will be censured for not having acceded to the requested removal at the first visit.

The operator should be provided with an abundant supply of forceps, including several not found at dental depots, with small and sharp beaks of various forms and sizes, for seizing deep-seated roots; lancets, screws, punches, and a dozen or more elevators of different forms, and most of them with small thin points to penetrate between roots and the walls of the sockets.

It is proper before performing this operation to attempt to dispel the fears of timid patients, but deception should never be practiced. Respectful suggestions should be kindly received, but dictation ought never to be tolerated. The patient who absolutely persists in having a tooth extracted in the manner he desires should be summarily discharged.

In the removal of teeth the dentist should not become excited; and even in difficult cases he should keep cool. If disconcerted, although he may try to avoid manifesting it by word or look, the patient will understand and be unfavorably affected by it.

Every tooth to be extracted, and especially those much decayed, should be carefully examined, the manner of applying force decided on, as well as plan of proceeding if an accident occurs, the instruments are to be selected, and the ability of the patient to endure protracted pain, if necessary, is to be considered.

Some anæsthetic should be used in the extraction of teeth, either nitrous oxide in suitable cases, or an application to the gums.

Lancing the gums, a barbarous, and usually a very painful performance, is rarely necessary. If proper instruments are skillfully used gums not lanced will very seldom be lacerated in the removal of teeth.

With the head in the best position the tooth is to be seized as near the border of the alveolus as possible, unless from the extent of caries there is fear of fracture, when small pointed forceps may, if necessary, be passed between the sides of the roots and sockets, or they may be so placed as to get a hold on a root by breaking opposite sides of the alveoli; or a small elevator may be inserted deeply enough in the socket by the side of a root to raise it, or such other means can be employed as may be thought feasible.

A tooth should never be extracted in a hurry. Force should be exerted cautiously, and in the direction of least resistance, and the movements watched, with care taken to avoid losing the hold on the tooth or roots. By applying force gradually the shock to the patient, and danger from accidents, are lessened.

When a tooth is broken in an attempt to extract it the roots should be taken out if possible, in which there is seldom much difficulty with the necessary instruments and skill in their use, except in rare cases or an uncontrolable patient. Roots so left will often afford a chance to seize them or to remove them with elevators in a few months; while in numerous instances a small portion of a root left in its socket causes no subsequent annoyance.

In case of syncope, which very seldom occurs, cold water should be dashed in the face, a vial of aqua ammonia held to the nostrils one or two seconds, and the patient placed in a recumbent position with the head as low as the body, and if the dress is tight about the waist it is well to loosen it, which will usually be followed by restoration in a short time.

Troublesome hemorrhage rarely follows the extraction of a tooth, nor is it difficult to suppress. Styptics applied to the gums may sometimes be advisable, but they are useless in the sockets, pressure being the only reliable remedy. Pledgets of cotton saturated with cold or hot water—preferably the latter—packed closely in the socket, and a compress on them to make pressure when the maxilla are closed, with a bandage under the chin tied above the head, is the proper treatment, and will scarcely ever fail to stop the hemorrhage immediately. The compress may be dispensed with in a few hours, and the cotton removed in three or four days.

Treatment immediately after the extraction of teeth may be necessary. If there is severe pain applications of hot water, tincture of camphor, or any of the numerous anæsthetics may be employed. When these fail to afford relief, morphia, or a dose of whisky or brandy, may prove useful.

It is well to cut off with scissors prominent points of loose gums. If sharp edges of the alveoli are left they should be removed with cutting forceps or a file. When the sides of sockets of the molars are forced outward in extracting they should be pressed into normal position. Pieces of alveolar process broken loose should be taken away. If the gums are much diseased it is important to give a prescription for their treatment.

Dislocation of the inferior maxilla, an accident of very rare occurrence, is to be reduced by making pressure on the molars, or where they were, by the thumb, or by placing corks there, and exerting force under the chin upward and backward which will readily prove successful.

When all the teeth of one or both maxilla are removed the patient, unless substitues are now to be supplied, should be directed to subsist on soft food, recollecting that a milk diet maintains its ancient preeminence for edentate persons, to eschew dead animals entirely, and to eat very slowly moving the food about the mouth to insalivate it to have it in the best condition for the action of the digestive organs.

